# A Review on Macroscopic and Microstructural Features of Metallic Coating Created by Pulsed Laser Material Deposition

**DOI:** 10.3390/mi13050659

**Published:** 2022-04-22

**Authors:** Xinlin Wang, Jinkun Jiang, Yongchang Tian

**Affiliations:** School of Mechanical Engineering, Dalian Jiaotong University, Dalian 116028, China; jiangjinkundybala@163.com (J.J.); s45542568@163.com (Y.T.)

**Keywords:** laser material deposition, pulse wave laser, temperature field simulation, surface quality, microstructural features, corrosion behavior

## Abstract

Owing to the unparalleled advantages in repairing of high value-add component with big size, fabricating of functionally graded material, and cladding to enhance the surface properties of parts, the laser material deposition (LMD) is widely used. Compared to the continuous wave (CW) laser, the controllability of the laser energy would be improved and the temperature history would be different under the condition of pulse wave (PW) laser through changing the pulse parameters, such as duty cycle and pulse frequency. In this paper, the research status of temperature field simulation, surface quality, microstructural features, including microstructures, microhardness, residual stress, and cracking, as well as corrosion behavior of metallic coating created by pulsed laser material deposition have been reviewed. Furthermore, the existing knowledge and technology gaps are identified while the future research directions are also discussed.

## 1. Introduction

**Figure 1:**
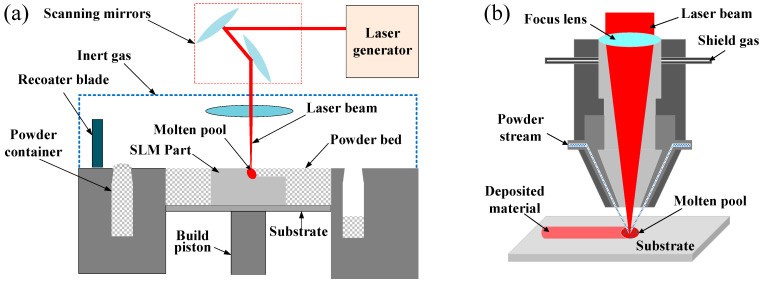
Schematic illustration of (**a**) powder bed fusion and (**b**) direct laser deposition LAM.

The conventional subtractive manufacturing process presents insufficient capability in the fabrication of complex structure and high value-added parts, because of high material removal rate, time consumption, and high cost during removing material from a large stock or sheet. On the contrary, laser additive manufacturing (LAM) can cover the shortage and create the final metal part because of the incremental layer-by-layer manufacturing by adding material [1,2,3]. Owing to the superiorities of excellent stability, high power density, and easy controllability, LAM is widely used in direct deposition of metal materials [4,5,6]. LAM mainly includes powder bed fusion (PBF) mode [7,8], in which the powder is preset in powder bed, and direct laser deposition (DED) mode [9], in which the powder would be delivered by inert gas into the molten pool. Powder bed fusion LAM (Figure 1a), such as selective laser sintering/selective laser melting, is more suitable for the parts with small size and complex structure. Meanwhile, direct laser deposition LAM (Figure 1b), such as laser material deposition (LMD), presents unparalleled advantages in repairing of high value-add component with big size, fabricating of functionally graded material, and cladding to enhance the surface properties of parts [10]. In LMD process, a high-powered laser beam is acted as the heating source to melt the substrate and create a molten pool. Meanwhile, the metallic powder carried by a flowing inert gas (such as Argon) is delivered into the molten pool [11]. The molten pool would capture the delivered powder and solidify after the laser beam move out that contributes to the increased volume of molten pool. Along the scanning path guided by the computer, the laser beam and the powder nozzle would move above the substrate. After the completion of depositing the first layer on the substrate following the scanning path, the laser cladding head upraised certain height in the z-axis increment to the new position to continue deposition of the next layer. Based on the first layer, the new layer would be deposited along the scanning path and create the metallurgical bonding with first layer. Similar process, in which the latter layer is sequentially deposited on the former, would be repeated to build a three-dimensional (3D) part layer-by-layer [12,13,14]. Due to the advantages such as high geometry freedom, low thermal inputting, high production flexibility, LMD is a competitive additive manufacturing technology in the areas of aerospace, aviation, die and mold, etc. [15,16].

In LMD process, the continuous wave (CW) laser, which is a common laser mode used presently, inputs relatively low heat into the substrate that attributes to the obtaining of high temperature gradient and high cooling rate during laser beam scanning compared to the conventional heating source such as plasma. Owing to the process feature of rapidly melting and solidification during the moving of laser beam, the refined microstructure and superior mechanical properties are created [17]. The typical solidification structure, including the planar crystal in the interface, dendritic crystal in the central region and cellular crystal near the top surface of deposition, would be presented. The deposition layer possess high hardness, high strength but low ductility [18,19]. However, the rapid melting and solidification would result in the residual stress, especially the tensile residual stress, which is a negative factor for the mechanical properties [20,21,22]. Researchers has made many efforts to optimize the LMD process and eliminate the unfavorable features such as low ductility and high residual stress. Among that, the pulsed wave (PW) laser, which is acted as the heating source to melt the substrate and powder, is proved to be a suitable method to control the melting process in LMD.

The working of CW laser is fairly simple where a continuous beam of light is emitted at an average power. However, based on Q-switching, mode-locking or pulse pumping methods, a PW laser produces high energy pulses. A pulsed laser, where lasers emitting optical power in the form of pulses at constant time intervals, means a fixed amount of energy for a specified duration [23,24]. The difference between the continuous and pulsed laser can be noticed in the average power and peak power. The continuous laser is a better option during a higher average power is required. However, compared to the continuous laser, the pulsed laser can produce higher peak power which attributes to the higher melting temperature and the improvement of surface finish during the same average power is used. Another important parameter to be understand is laser energy density, which is represented by the laser energy per unit area and generally expressed as J/cm^2^. The laser energy is related to the peak power and the duration time of laser. Therefore, in comparison with the continuous laser, the pulsed laser can produce higher energy density owing to the higher peak power. In addition, during the peak power is constant, the average power produced by pulsed laser is smaller compared to the CW laser mode. This is because the interval time between laser pulses enables a lower heat input into the substrate during the pulsed laser is used [24]. Furthermore, the interval time provides the blanking time for the cooling and solidification of the molten pool. So the parts with more refined microstructure and higher hardness can be produced. Through changing of the pulsed laser parameters such as pulse width, interval time and frequency etc., the controllability of the laser energy can be improved with the application of pulsed laser. The thermal history and temperature distribution can be regulated by controlling the laser energy emitted by the pulsed laser.

The capability of pulsed laser to modifying the thermal history and solidification process of molten pool has drawn researchers’ much attention. Some investigations about the comparison of LMD process between the CW and PW laser as well as the effect of pulse laser parameters, such as pulse width, duty cycle, and pulse frequency, on the microstructure feature and mechanical properties have been conducted. The deposited material refers to Fe-based alloy, Co-based alloy, Ni-based alloy, titanium, ceramic and so on. Table 1 provides a summary of some common materials that have been investigated for the cladding and deposition by pulsed laser material deposition. With the changes of the deposited materials and pulsed laser parameters, the deposition would present different characteristics. This article provides an overview of the macroscopic and microstructural features of metallic coating created by pulsed laser material deposition.

## 2. Temperature Field Simulation and Thermal Analysis

With a view to evaluate the thermal profile during the LMD process, temperature field simulation and thermal analysis are usually conducted. The thermal profile and temperature distribution during the deposition are closely related to the process parameters which determines the laser energy inputting and the cooling rate of molten pool. The laser mode of PW, which can control the laser energy inputting as well as the interaction among the laser energy, powder, and substrate by changing the parameters of pulsed laser, has a significant effect on the thermal profile during the deposition process [48,49].

In the LMD process, the Gaussian conical heat source is recognized as the representative of laser beam. Thus a Gaussian conical heat source [50], which can be mathematically represented by Equation (1), generally was used as the thermal loading during the simulation of LMD process [51,52].

(1) $$q\left(x,y,z\right)=\frac{9Q_{0}}{\pi \left(1-e^{-3}\right)\left(z_{e}-z_{i}\right)\left(r_{e}^{2}+r_{e}r_{i}+r_{i}^{2}\right)}exp\left(-\frac{x^{2}+y^{2}}{r_{0}^{2}\left(z\right)}\right)$$

where, *Q*_0_ is the heat flux and *Q*_0_ = *ηP*, *P* is the laser beam energy, *η* is the efficiency value, and *ηP* represents the heat flux. *r*_0_ is the heat distribution coefficient and can be represented by Equation (2),

(2) $$r_{0}\left(z\right)=r_{i}+\left(r_{e}-r_{i}\right)\frac{z-z_{i}}{z_{e}-z_{i}}$$

where *r_e_* and *r_i_* represent the maximum and minimum radius, respectively.

Owing to the existing of laser-on and laser-off stage in the PW laser mode, the constant about time should be defined. K. Yang et al. defined a constant about time (δ(t)), as represented by Equation (3), and imported into the heat conduction equation to simulate temperature field in the LMD process with PW laser mode [27].

(3) $$\delta \left(t\right)=\{\begin{array}{c} 0\;\;\;\;\;\;\;\;\;\;\;\;0\leq t\leq T_{pulse}\\ 1\;\;\;\;\;\;\;\;T_{pulse}<t\leq T_{cycle} \end{array}$$

(4) $$\delta \left(t+T_{cycle}\right)=\delta \left(t\right)$$

where *T_pulse_* represents the pulse width and *T_cycle_* represents the cycle period of one pulse. *T_pulse_* and *T_cycle_* can be calculated by pulse frequency *f_pulse_* and the duty cycle *D_u_* as follow:

(5) $$T_{cycle}=1/f_{pulse}$$

(6) $$T_{pulse}=D_{u}T_{cycle}$$

**Figure 2:**
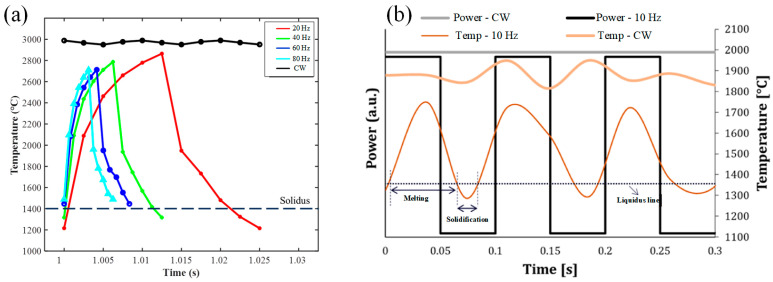
The temperature variation of molten pool by CW and PW temperature field based on (**a**) simulation (Reprinted from ref. [27], copyright (2021), with permission from Elsevier) and (**b**) experimental results where the peak power is constant (Reprinted from ref. [32], copyright (2020), with permission from Elsevier).

With the comparison of CW laser mode, the study reported by Z. Yang et al., in which the duty cycle was 50%, showed the peak temperature variation in one pulse period with the difference of pulse frequency as shown in Figure 2a. During the LMD process of high-nitrogen steel (HNS), the peak temperature of the melt pool created by CW laser mode was about 2900 °C and remained almost stable. Under the PW laser mode regardless of the pulse frequency, the temperature of molten pool increased dramatically with a gradual decline of growth rate when the laser was ON, and then, decreased dramatically when the laser was OFF. With the increase of pulse frequency from 20 Hz to 80 Hz, the maximum temperature decreased from 2860 °C to 2690 °C and the minimum temperature increased from 1210 °C to 1480 °C. Under the pulse frequency of 60 Hz and 80 Hz, the minimum temperature of molten pool exceeded the solidus temperature (1400 °C) of HNS that meant the molten pool kept continuous during the LMD process. Thus, the fish-scale patterns were not observed on the surface morphology of single-track clad that also proofed by the experiments. The pulse frequency of 60 Hz was the critical value which decided whether the molten pool remained continuous or not [27]. Therefore, the pulse frequency should be larger than the critical value to assure the continuous molten pool.

**Figure 3:**
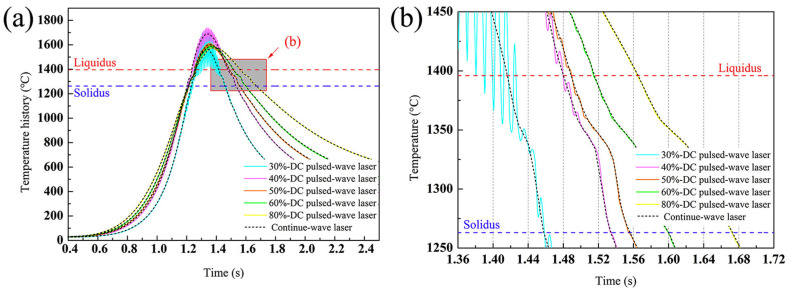
(**a**) Temperature history at the point that is 120 μm above the bonding interface in the depositions with different duty cycles, and (**b**) Enlargement of the shaded part in (**a**). (Reprinted from ref. [33], copyright (2020), with permission from Elsevier).

The thermal history has significant effect on the cooling rate and then the dendrite spacing and microstructure. The influence of cooling rate on the dendrite spacing could be described by the following equation [33,53]:

(7) $$\lambda =C\cdot \varepsilon^{-m}$$

where λ is the dendrite spacing, C and m represent positive constants determined by the material, ε is the cooling rate. Based on the Equation (7), it can be concluded that the dendrite spacing would increase with the increase of cooling rate. According to the simulation results about deposition of non-weldable nickel-based K447A alloy, Z. Zhang et al. pointed that the temperature history under the PW laser was more vibrational compared to that created by CW laser in the high-temperature region as shown in Figure 3. The vibrational amplitude reduced with the increase of duty cycle and the temperature curve with the duty cycle of 0.8 closed to consistent with the curve under the CW laser. The cooling rate at around 1370 °C and the dendrite spacing of depositions under different parameters were shown in Table 2. The cooling rate induced by PW laser presented higher than that by CW laser. However, the difference became smaller with the increase of duty cycle and the cooling rate under the duty cycle of 0.8 became same with that of CW laser owing to the consistent temperature curve. The dendrite spacing increased from 4.87 μm to 6.86 μm with the increase of duty cycle from 0.3 to 0.8 [33].

The actual thermal profile during the deposition process can be monitored by the infra-red camera and the thermocouples. With the using of CW laser mode, the measured average temperature could reach about 1900 °C during the Inconel 718 deposition was created by the laser powder of 300 W as shown in Figure 3b. However, the measured temperature presented slight oscillations owing to the powder sparks which were caused by the turbulence and expulsion of molten pool. During the PW laser with the pulse frequency of 10 Hz was used, the temperature presented periodic variation between the maximum value of 1700 °C and the minimum value of 1300 °C that attributed to the “heart-beat” behavior and thus the local thermal cycles in the molten pool. The lower temperature and local thermal cycles were obtained by using the pulsed laser within each deposited layer. It is worth noting that the temperature drop to 1300 °C, which was lower than the solidus temperature of 1350 °C during the PW laser with the pulse frequency of 10 Hz was used. Compared to the pulse frequency of 100 Hz and 1000 Hz, the measured temperature created by pulse frequency of 10 Hz led to low laser energy inputting, low heat accumulation, and high cooling rate which resulted in the measured temperature decreased under the solidus temperature. Therefore, the molten pool was subject by solidification-melting cycles. In addition, during the pulse frequency of 100 Hz was used, the temperature reached the value which was slightly higher than the solidus temperature when the laser was set on OFF. Thus, during the Inconel 718 deposition was created by the laser powder of 300 W, the pulse frequency of 100 Hz can be recognized as the transition value which determined whether the temperature down to the solidus temperature and the molten pool remains continuous or not [32].

Based on the reported results obtained by simulation and actual experiment, the temperature created by the PW laser would be lower than that by CW laser because of the existing of the interval time when the laser is set on OFF. Thus, lower heat accumulation and higher cooling rate of molten pool could be obtained. As for the PW laser mode, there is an approximate pulse frequency which can be recognized as demarcation line to distinguish the status of molten pool during the deposition process.

## 3. Surface Quality

In the LMD process, the process parameters, such as laser power, powder feed rate, z-increment, shielding gas flow and so on, govern the temperature field of molten pool then consequently affect the Marangoni flow and the surface quality [54,55]. Marangoni flow of the molten pool, which is closely related to the temperature gradient, would happen when the material absorbs the inputted laser energy and melt. Owing to the Gaussian conical heat source, the energy density near the middle of molten pool is high and it is low near the edge that contributes to the occurrence of temperature gradient and then the surface tension gradient. The Marangoni flow is mainly caused by the different of surface tension in the different region of molten pool. The high energy density corresponds to high temperature and low surface tension. Thus, the liquid metal would flow from the central region to the edge of the molten pool [56,57,58]. The surface quality, which is reflected by the surface roughness, is strongly linked with the intensity of Marangoni flow and vapour recoil pressure. According to the driving force of the recoil pressure and the Marangoni force, once the local momentum exceeds the pressure produced by the surface tension, the molten materials in the molten pool are ejected that is not conductive to the surface quality [59,60]. However, the strong Marangoni flow contributes to the aggravation of the surface disturbance of molten pool which is beneficial to mix and melt the powder particles within the molten pool and was found to be a vital parameter in decreasing the surface roughness [61].

**Figure 4:**
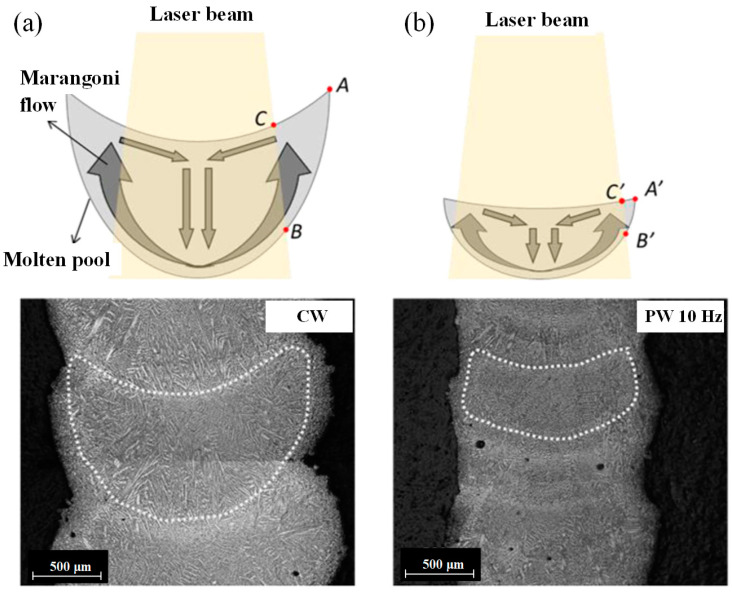
Marangoni flow within the cross-section of the IN718 thin walls when (**a**) CW and (**b**) 10 Hz PW laser are used. (Reprinted from ref. [32], copyright (2020), with permission from Elsevier).

Compared to the CW laser, with using of PW laser in the LMD process, the molten pool can be modified by the high surface disturbance of molten pool which improves the mixing action of powder into the molten pool and the melting efficiency of captured powder. K. Shah et al. deposited Inconel 718 powder on a Ti-6Al-4V substrate by using CW and PW laser. The experimental results presented an inverse relationship between the surface disturbance of the melt pool and the surface roughness of the part. Using the PW laser increased the mean surface disturbance by experiencing high peak power [61]. A. Pinkerton and L. Li pointed out that increasing the duty cycle, up to the boundary case of a CW laser, increased the surface roughness through conducting the multiple-layer 316 L steel deposition with the CW and PW laser [62]. In addition, as reported by S. Imbrogno et al., the width of molten pool created by different wave of laser had significant effects on the side surface quality of thin-wall deposition. As shown in Figure 4a, owing to the high laser energy inputted by CW laser, the width of molten pool was bigger than that created by PW laser with the pulse frequency of 10 Hz shown in Figure 4b. The temperature in region ABC was lower than the central region of molten pool because this region was not directly irradiated by laser beam that resulted in most of heat in this region could be conducted by the melted material. During the PW laser was used, the area of region A′B′C′ was smaller that contributed to the improvement of side surface quality. Moreover, compared to the CW laser, the less U-shaped molten pool, which was beneficial for reducing the waviness effect on the side surface, was presented when the PW laser was used because of the less energy per time unit and the lower temperature of molten pool [32]. Thus, the side surface quality of thin-wall was improved by using the PW laser. M. Gharbi et al. deposited the Titanium powder on the Ti-6Al-4V alloy with different laser wave and investigated the influence of different PW laser parameters on the surface quality. They pointed out that the surface finish of depositions, in which the average rough values Ra was 3 μm, was obtained using the PW laser owing to the decreased thermal gradients and Marangoni flow of the molten pool. However, compared to the PW Gaussian laser, the using of top-hat laser not presented further improvement to the surface quality [46].

## 4. Microstructures

**Figure 5:**
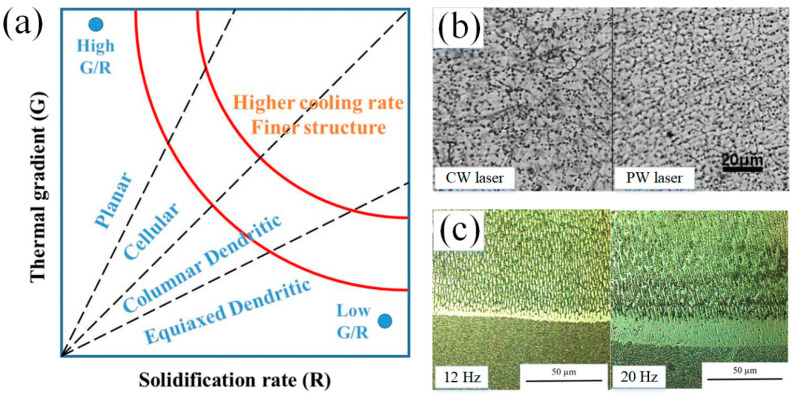
(**a**) Solidification map established by solidification parameters (Reprinted from ref. [27], copyright (2021), with permission from Elsevier); (**b**) Optical micrographs of CW and PW laser cladding Fe-based depositions (Reprinted from ref. [25], copyright (2014), with permission from Elsevier); (**c**) OM images of the intermixing zone of Inconel 713 deposition with a laser frequency of 12 Hz and 20 Hz (Reprinted from ref. [37], copyright (2021), with permission from Elsevier).

The inputted laser energy has significant effect on the thermal history which can dominate the microstructure features of depositions such as morphology and grain size as shown in Figure 5a. The solidification rate of molten pool, the temperature gradient at the solid liquid interface (G), and the ratio of cooling rate to thermal gradient (R) determine the solidified microstructure. The G/R would affect the solid-liquid interface shape and the G × R, which means the cooling rate, would affect the grain size [4,63]. The G/R is large during the early stage of LMD process that contributes to the occurrence of columnar crystal. Owing to the decrease of G/R with the continuous process of LAM, the dendrite crystal tends to form [64,65,66].

Although the nominal laser power is same, the PW laser heat input would be lower than that created by the CW laser owing to the existing of duty cycle. The increase of the heat input reduces the cooling rate and increases the grain size [67]. The PW laser with higher cooling rate in molten pool can result in the bigger supercooling degree which contributes to the increase of nucleation rate and nuclei number. In addition, owing to the existing of intermittent, the PW laser would urge the occurrence of thermal shock effect on the molten pool that prevents the reuniting of nuclei after separating out from the molten pool. Also, the intermittent provides the blanking time for the molten pool to solidify that results in the shorter solidification time of the molten pool by PW laser than that created by CW laser. Furthermore, compared to the CW laser, the PW laser would reduce the heat accumulation and increase cooling rate that results in the grains have not enough time to grow along with the orientation driven by the heat source. The lower pulse frequency means bigger interval time during the laser is set on OFF that contributes to longer solidification time for the melted material and higher cooling rate. This has been verified by the experiment results about Inconel 718 deposition fabricated by pulse laser with the frequency of 10 Hz through comparing to the microstructures created by the pulse frequency of 100 Hz and 1000 Hz [32]. Therefore, the microstructure in PW deposition layer was refined significantly. During the coagulation process in the molten pool, the deposition underwent nucleation. The decrease of gain size contributed to the increase of nucleation base number and nucleation rate. Therefore, the PW laser produced the refined microstructure that was verified by researchers based on the experimental results [32]. For example, the research reported by H. Zhang et al. showed the significant refined microstructure in Fe-based coating on low carbon steel though using the PW laser that concluded by the CW and PW laser deposition’s average grain size of 11.68 μm and 6.86 μm, respectively as shown in Figure 5b [25]. Decreasing the heat input though reducing laser frequency could prompt the transformation of from columnar grains to the equiaxed grains with smaller size as shown in the Stellite 31 coating on the Inconel 713 substrate as shown in Figure 5c [37]. For the Ti-based coating, K. Xiang et al. fabricated the CoNiTi medium-entropy alloy depositions on the pure Ti substrate and obtained the good metallurgical bonding between the depositions and substrate using the pulsed LMD. The deposition consisted of dendritic-interdendritic microstructure. The bonding zone, which was followed by the heat affected zone with irregular-shaped bulk grains was comprised of acicular fine grains with an average width of ~320 nm [44]. The Ti coating obtained by C. Wang et al. presented disorderly distributed β phase and fine martensitic lath [47].

**Figure 6:**
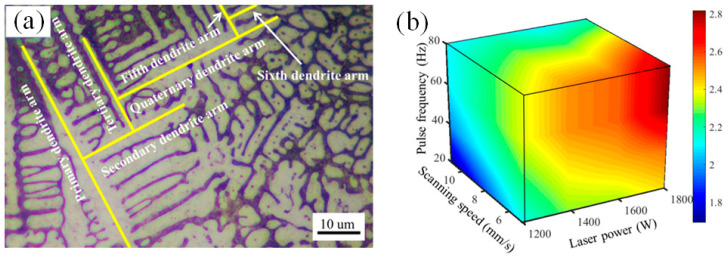
(**a**) From primary- to sixth- dendrite arm, and (**b**) Secondary dendrite arm spacing (SDAS) values map for single track deposition of high-nitrogen steel. (Reprinted from ref. [27], copyright (2019), with permission from Elsevier).

In addition, the secondary dendrite arm spacing (SDAS) was usually chosen as the representation of microstructure evolution. The SDAS values decreased along the direction from bottom to top of the deposition layer due to the different cooling rate in the different region of molten pool. The SDAS would increase with the increase of laser power and reduction of scanning speed because of the increased heat inputting and decreased cooling rate of molten pool. The variation of PW laser parameters exactly affects the heat inputting and the SDAS. Z. Yang et al. pointed out that the SDAS in high-nitrogen steel deposition increased dramatically with the pulse frequency increased from 20 Hz to 60 Hz during the other parameters kept constant. However, during the pulse frequency exceeded to 60 Hz, the effect on SDAS became minimal. An interesting phenomenon was presented that a sixth dendrite arm was observed in the pulsed LAM samples as shown in Figure 6a. It turned out that that as long as there were sufficient spaces and large enough solidification rates, new branches can continuously grow from the primary dendrite arms. In addition, the SDAS value map was plotted as a function of laser power, scanning speed, and pulse frequency as shown in Figure 6b [27].

**Figure 7:**
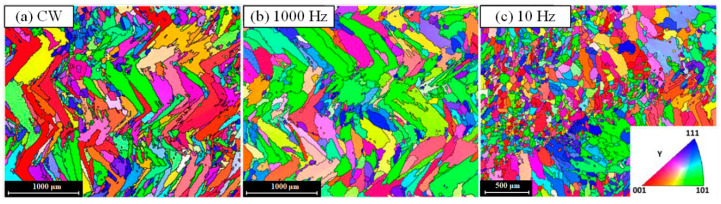
EBSD map of the longitudinal section zx plane during using of (**a**) CW, (**b**) PW with the frequency of 1000 Hz and (**c**) PW with the frequency of 10 Hz (Reprinted from ref. [32], copyright (2020), with permission from Elsevier).

Furthermore, owing to the change of heat inputted and laser energy with the different wave of laser as well as the parameters of PW laser such as pulse frequency, the orientation of the grains, which is closely related to the heat source movement, would be affected. In general, during the CW laser is used, the molten pool would be stable and continuous owing the constant laser energy inputting. As shown in the investigation about Inconel 718 thin-wall deposition by LMD reported by S. Imbrogno et al., the microstructure of the longitudinal section of deposition, which was created by CW laser and PW laser with pulse frequency of 100 Hz and 1000 Hz shown in Figure 7a,b, presented epitaxial growth and the growing direction was determined by the heat source movement. When the pulse frequency of 10 Hz was used, the obvious discrete molten pool boundaries was observed and the growing of dendrites oriented to the center of each discrete molten pool as shown in Figure 7c [32]. G. Muvvala et al. indicated that the using of PW laser resulted in stacks of columnar dendrites grow along different orientation owing to the periodic remelting and solidification of irradiated volume [68]. A. Farnia et al. deposited the Stellite 6 coating on the low carbon ferritic steel with the pulse frequency ranged from 1 to 1000 Hz, pulse duration ranged from 0.2 to 20 ms, and pulse energy ranged from 0 to 40 J. The results illustrated that the pulse traces can represent the solidification fronts. The orientation of the grains showed perpendicular to the solidification front. Based on the elongation and orientation of grains near the middle region of coating, the results indicated that the shape of solidification front during the PW laser was used was similar to that produced by the CW laser with a double scanning speed. In the pulsed LMD process, the consecutive pulse resulted in the periodical repetition of grain orientations on the longitudinal section of coating [36]. Therefore, the pulse frequency plays an important role in not only the microstructure refinement but also the growing orientation of grains.

## 5. Microhardness

**Figure 8:**
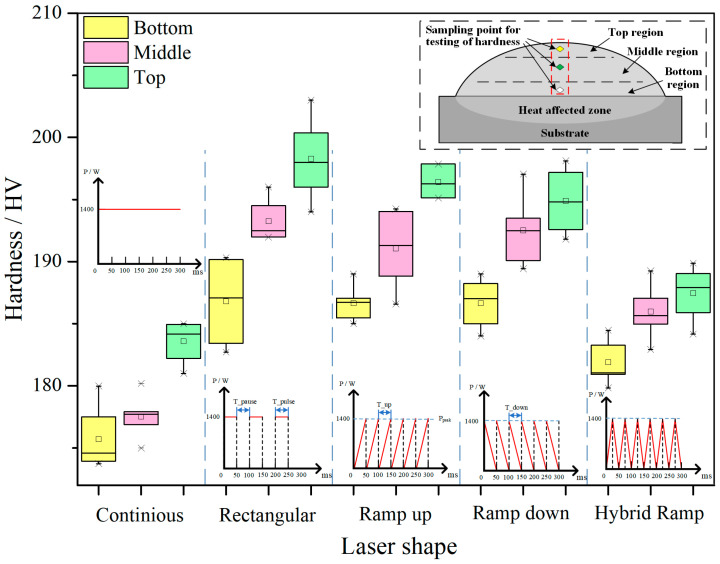
Microhardness at different regions of AISL316L deposition produced by different laser shape. (Reprinted from ref. [29], copyright (2020), with permission from MDPI.).

Based on the classic Hall-Petch equation $\sigma_{y}=\sigma_{0}+{kd}^{-1/2}$ [69,70], where $\sigma_{y}$ is yield or tensile stress, $\sigma_{0}$ is a friction stress which is a constant stress for steel stress during dislocations move on the slip plane, k is the stress concentration factor which is reflected by the Hall-Petch slope, d is the average grain size The yield and tensile stress decrease with the increase of reciprocal root of the grain size that means the yield and tensile strength positively correlated to the grain size. H. Zhang et al. compared the microhardenss of TiC-VC reinforced Fe-based cladding created CW and PW laser. The average harnesses of CW and PW cladding layers are 950 HV_0.2_ and 1160 HV_0.2_, respectively. The results were consistent with grain size measurement results where the CW and PW laser cladding layers’ average grain sizes are 11.68 μm and 6.86 μm, respectively. That shown the dispersion strengthening effect, in which the carbides refinement is caused by using the PW laser, and improvement of hardness [25]. The effect of grain size on strength was identical to that on the hardness [71,72]. S. Imbrogno et al. deposited Inconel 718 thin-wall by CW and PW laser, and pointed out that the hardness measured on the PW depositions was higher than that on the CW samples because of the lower heat accumulation, higher cooling rate, and smaller the primary dendrite arm spacing (PDAS). In addition, the hardness gradually decreased from the bottom to the top of depositions during the PW laser was used. However, the hardness on the sample produced by CW presented a constant distribution and a rapid drop only on the top part [32]. A. Khorram investigated the effect of parameters of PW laser on the Stellite 31 coating by LMD in which the Inconel 713 was chosen as the substrate. Based on the experimental results, they pointed out that the hardness increased almost linearly with the decrease of dilution ration which was determined by the reduction of pulse width and laser frequency [37,73]. X. Wang et al. investigated the effect of different pulsed laser shape, including continuous, rectangular, ramp up, ramp down, and hybrid ramp, on the hardness of AISI316L deposition. They pointed out that the deposition created by rectangular laser shape presented highest hardness because of the minimum value of grain size caused by the high cooling rate as shown in Figure 8 [29].

The improvement of hardness sometimes contributes to the formation of strengthening structure and hardening phase. The deposition of CoNiTi medium-entropy alloy created by PW laser reached to 571 ± 46 HV which was about five times of the hardness in the substrate (114 ± 4 HV) owing to the formation of solid-solution hardening BCC phase and second-phase hardening from the Ti_2_Ni and Ti_2_Co type intermetallic compounds [44]. C. Wang investigated the hardness of Ti coating created by PW laser on the Ti-6Al-4V substrate in which consisted of α grains with the grain size of 4.7 μm. The lath structure martensite with the size of 0.86 μm was presented in the coating with fine grains after pulsed laser clad that contributed to the improvement of hardness. During the pulse frequency of 16 Hz and pulse width of 13 ms were set in the deposition process, the hardness Stellite 31 coating with the value of 482 ± 10 HV, which is markedly higher than the hardness of Inconel 713 substrate with the value of 355 ± 3 HV, was presented. The improvement of hardness in the deposition contributed to the creation of Co solid solution phase and Cr_7_C_3_, Cr_3_C_2_, W_2_C, and WC_x_ carbides [37]. During the pulse frequency was 60 Hz, H.Yan et al. deposited Co-based coating on the copper alloy substrate. The coating presented high hardness with the value about 478 HV which was about five times that of the copper substrate (92 HV) that contributed to the formation of carbide and intermetallic hard phases [41]. W. Wang et al. conducted the repairing of BT20 titanium alloy with the pulse width of 12 ms and the frequency of 4 Hz and obtained the coating without defects. The hardness of deposition presented even mean value (about 450 HV) which was slight higher than that of substrate (about 370 HV). During the LMD process, the transformation of “β→α′” martensite, in which the β stable elements, such as Mo and V, soluted into α′ phase, attributed to the improvement of hardness [74]. Owing to the occurrence of dilution which was necessary for the formation of metallurgical bonding between the substrate and depositions, the hardness was affected. Through cladding the 75Cr_3_C_2_ + 25 (80Ni20Cr) powder on the Inconel 718, A. Khorram et al. pointed out that with the increase of pulse frequency and pulse width as well as the decrease of laser scanning speed, the dilution ratio increased that contributed to the decrease of concentration of chromium carbides in the deposition and the formation of eutectic structure. Therefore, hardness in the deposition reduced. However, based on the optimum parameters, including laser frequency of 20 Hz, pulse width of 12.9 ms, and laser speed of 5.43 mm/s, the hardness of deposition was improved with the value of 1050 HV which was about 2.5 times that of the substrate that attributed to the abundant chromium carbide (Cr_7_C_3_) and refined eutectic structure (γ + Cr_7_C_3_) [75]. G. Muvvala et al. indicated that the increasing of cooling rate contributed to the decrease of elemental segregation, formation of Laves phase and γ matrix size that resulted in the hardness improvement of Inconel 718 deposition [68].

## 6. Residual Stress and Crack

**Figure 9:**
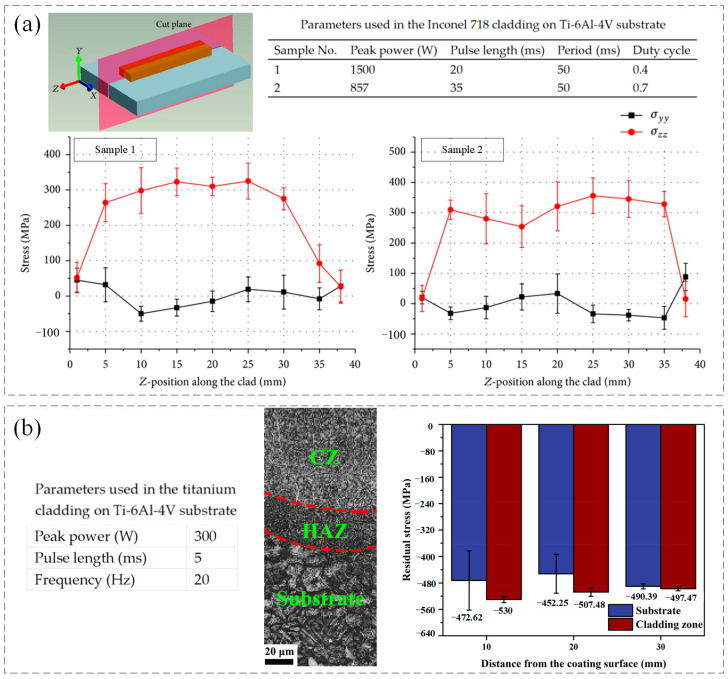
(**a**) Residual stress of Inconel 718 cladding creased by different parameters of PW laser (Reprinted from ref. [31], copyright (2014), with permission from Hindawi.); (**b**)Residual stress of substrate and coating zone. (Reprinted from ref. [47], copyright (2021), with permission from Elsevier).

The LMD process is accompanied by the repeated rapid heating and cooling of molten pool. Owing to a heterogeneous response of heat conduction and heat dissipation, the high residual stress, which is a domain drawback for the corrosion, fracture resistance, and fatigue performance, is easily to produce in the deposition itself and at the interface between clad and substrate areas as a result of the fast-cooling rates and the difference in thermal expansion coefficients [76,77,78,79,80]. The residual stress would be generated by the constrained thermal shrinkage, which is caused by the transient temperature gradients, the different coefficient of thermal expansion between the substrate and deposition, and the changes in specific density caused by transformation of solid phase [18,81,82]. The heat inputting affects the cooling rate which has negative correlation to the square of the melt pool length [83]. Considering the mechanical factors, the cooling rate causes the thermal strains and increases the crack initiation rate. Compared to the CW laser, the PW laser, which can result in the high cooling rate, can increase the crack resistance that verified by K. Shah [31]. In addition, the residual stress increases with the reduction of pulse length and duty cycle as shown in Figure 9a [31,84].

**Figure 10:**
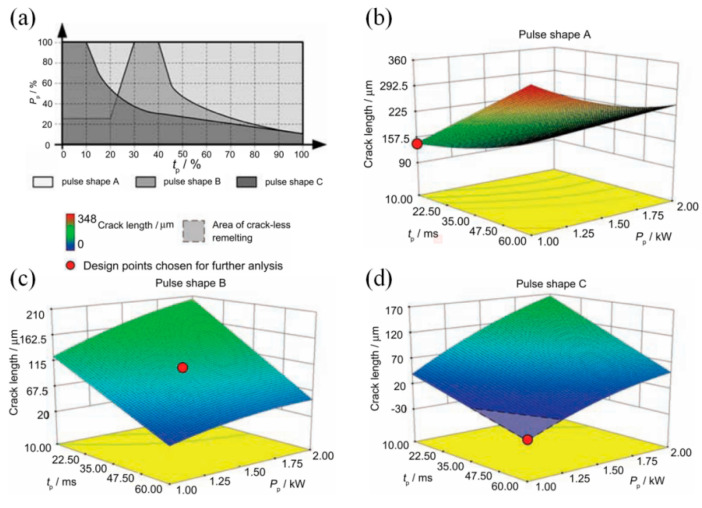
(**a**) Laser pulse shapes, and the 3-D RSM model plots of crack length in remelted spots during the (**b**) pulse A, (**c**) pulse B, and (**d**) pulse C was used. (Reprinted from ref. [96], copyright (2011), with permission from Elsevier).

In the LMD process, both macro- and micro-cracks may occur in the depositions. The macro-cracks, which are known as solidification cracks, often are generated near the solidification temperature range [85]. They are caused by the insufficient supplement of liquid metal when the consolidation and contraction process suddenly release stress after the increased thermal stress discharge from the rapid cooling process [86,87]. Micro-cracks mainly include phase interface cracking, slip zone cracking, and grain boundary cracking. They are usually caused by uneven local slip and micro-cracking of metal materials. In addition, small cavities, uneven crystal grains, and impurities produced in the LMD process are also the inducements for the micro-cracks [88,89]. It can be concluded that the crack is affected by the metallurgical factors, which is mainly concern phase relationship, and mechanical factors, which are related to stress behavior [31,90]. As for the metallurgical factors, the experimental results indicated that the changing of parameters was insufficient to avoid the generation of the brittle phases in the Ni-based deposition [31]. The LMD process undergone a high cyclic heating and cooling regime and the concentrated moving heat source made the LMD process vulnerable to thermal stresses which were the source of crack formation [91,92]. The tensile stress was unfavorable for the mechanical properties of parts because it decreased the fatigue life and tensile properties of the depositions. The comprehensive residual stress was beneficial for improving the fatigue and wear resistance of parts owing the effects of forbidding the initiation and propagation of cracks [93,94,95]. As shown in Figure 9b, compared to the Ti-6Al-4V substrate with the residual stress of −471.75 MPa, the residual stress in Ti coating created by PW laser reached to −511.65 MPa that contributed to the decreased susceptibility to crack and high resistance for crack generation and propagation [47]. Based on the pulse shaping, M. Pleterski conducted the laser cladding with wire on the AISI D2 (EN X160CrMo12-1) tool steel. Three types of laser shape, which including rectangular (shape A), ramp-down (shape B), and ramp-up-down (shape C), were used as shown in Figure 10. When the laser peak power was set between 1 and 1.5 kW and pulse duration ranged from 30 to 60 ms, the deposition created by shape A and B presented obvious cracks but the cracks under the shape B were shallower compared to those exhibited under shape A. However, no cracks were observed in the sample deposited by the shape C that contributed to the relatively slower cooling rate which was affected by the parameter setting [96].

During the cooling of molten pool in the LMD process, the shrinkage created by solidification of liquid phase and the deformation of solid structure produce the driving strain which attributes to the decrease of pressure in the liquid film and then the propagation of cracks. The driving strain increases with the increase of the dendritic spacing of the microstructure which is affected by the laser parameters. A void, which provides favorable condition for the propagation of cracks, would form when the pressure decreases below a cavitation pressure after the crack nucleates [33]. In addition, the cavitation pressure changes with the progress of solidification that can be described with the following equation [97,98]:

(8) $$P_{crit}=\frac{2\gamma_{sl}}{h}$$

where *h*, which has been widely recognized as the main influence factor, represents the thickness of the liquid film, $\gamma_{sl}$ represents the surface tension which is closely related to the temperature. With the increase of *h*, the crack becomes easier to occur in the liquid film between the adjacent dendrities. According to the surface penetration test of non-weldable nickel-based superalloy deposition reported by Z. Zhang’s work in which the pulse frequency was constant with the value of 10 Hz, evident cracks were observed during the duty cycle ranged from 0.6 to 0.8, and the deposition under the duty cycle ranged from 0.3 to 0.5 presented crack-free. The increase of duty cycle resulted in increasing of dendritic spacing and then bigger driving strain created by the shrinkage and deformation. The depositions with bigger dendritic spacing had weaker resistance to cracking and presented obvious cracks. In addition, the bigger duty cycle was used, the bigger width of dendrites and bigger dendrite spacing was caused by the smaller cooling rate [33]. Thus, the increase of duty cycle was not conductive to preventing from the propagation of cracks that also has been verified by the investigation of A. Odabsi et al. in which more evident and longer cracks were observed during the dendrite spacing increased from 1.06 μm to 2.3 μm [99]. Another way to preventing from the formation and propagation of cracks is preheating the substrate. J. Yin et al. obtained the defect-free Ni-Cr-Si coating on the copper substrate preheated with the preheating temperature of 700 °C [100]. With preheating of the copper substrate to 300 °C, Y. Zhang et al. deposited nickel based alloy coating which was free of cracks and composed of γ-nickel solid solution dendrites as well as some quantity of carbides and silicides [101].

## 7. Corrosion Behavior

The corrosion process, which is a gradual degradation process of material by chemical or electrochemical reaction with their environment, transforms a refined metal into a more chemically stable form such as sulfide, hydroxide, or oxide [102,103,104,105]. The corrosion resistance sometimes is a vital performance index to reflect the mechanical properties of the deposition by LMD when the working condition is poor such as high temperature, high pressure, corrosive atmosphere and so on. The solidified microstructure, which can be significantly affected by the laser energy inputting such as the pulse frequency in PW laser mode, determines the corrosion behavior of deposition by LMD. By using the low-pulse frequency, the refined solidified microstructure can be obtained. And the small grain size is beneficial to the corrosion behavior. The high density of grain boundaries, which is accompanied with the refinement of grains, contributes to the formation of metal passivation films followed by chloride ions [106]. The passivation films thickness is an important parameter of corrosion performance and is proportional to the corrosion resistance that have been demonstrated by many scholars [107,108].

**Figure 11:**
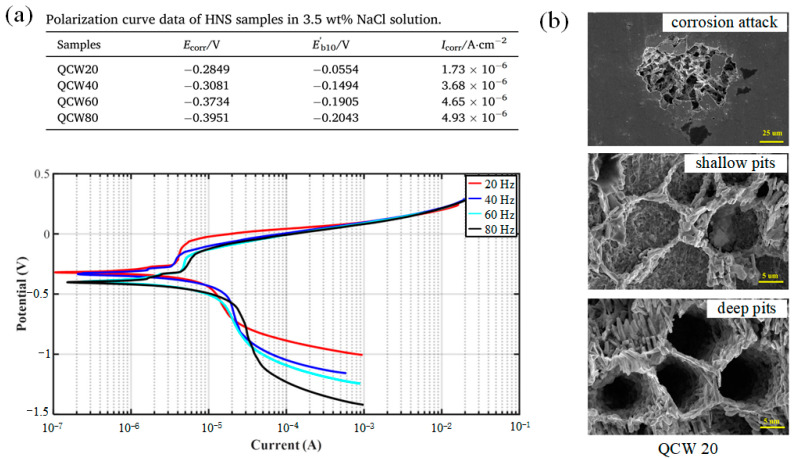
(**a**) Polarization curves of high-nitrogen steel (HNS) samples under various pulse frequencies, and (**b**) SEM images (2000×) of the QCW20 sample after the potentiodynamic polarization experiments. (Reprinted from ref. [27], copyright (2021), with permission from Elsevier).

In addition, the micro-porosity, which is related to the entrapment of gas in the molten pool, the unstable of melt flow behavior, and the local overheating of the molten pool, is also an important factor affecting the corrosion preformation of depositions [27]. However, except for the mentioned above formation mechanism, there will be other basic reason for the formation of micro-porosity during some material is used. For example, the root cause of the formation mechanism in the Inconel 718 depositions using PW laser with high frequency can be regarded as Nb segregation and the hydrogen segregation affected the micro-porosity in the aluminum alloys [109,110]. In addition, the influence factor of micro-porosity in the high-nitrogen steel depositions can be regarded as the nitrogen segregation. The high cooling rate contributed to the decrease of diffusion time of element that prevented the element segregation as proved by the macro-micro couple model developed by Xie et al. [50]. The high cooling rate of molten pool provided less time for pore growth and element diffusion that contributed to the improve of corrosion resistance. As shown in the research reported by K. Yang, the high-nitrogen steel depositions, which was created by PW laser with pulse frequency of 20 Hz, presented the most outstanding corrosion resistance with the lowest corrosion density of 1.73 × 10^−6^ A cm^−2^, the highest corrosion potential of −0.2849 V and pitting potential of −0.0554 V compared to the other four samples with pulse frequency of 0 Hz, 40 Hz, 60 Hz, 80 Hz, respectively as shown in Figure 11a. In addition, two completely different corrosion morphologies, which are shallow and deep pits as shown in Figure 11b, in QCW20 sample were presented. In the areas where the pitting occurs, the different dendrite morphology and micro-porosity size in the cross-sections resulted in distinct corrosion morphology. The micro-porosity content of depositions increased with the increase of pulse frequency and decrease of the cooling rate [27].

## 8. Conclusions

This review addresses the research status of temperature simulation, surface quality, microstructures, microhardness, residual stress, cracking, and corrosion behavior of metallic coating created by pulsed laser material deposition. The main conclusions are drawn as follows:

(1) According to the simulation and experimental results about temperature field during the LMD process, the using of PW laser would contribute to the lower heat accumulation and higher cooling rate of molten pool. Under the condition of PW laser, the minimum temperature of molten pool sometimes would be lower than the solidus temperature of the material that means the molten pool can not keep continuous during the LMD process. Thus, the fish-scale patterns would be observed. For example, the pulse frequency should be larger than the critical value to assure the continuous molten pool. The thermal history has significant effect on the cooling rate and then the dendrite spacing and microstructure.
(2) With using of PW laser in the LMD process, the molten pool would be modified by the high surface disturbance of molten pool created by the strong Marangoni flow that improves the mixing action of powder into the molten pool, the melting efficiency of captured powder, and thus the surface quality.
(3) The heat input of PW laser would be lower than that created by the CW laser owing to the existing of duty cycle that contributes to the increase of cooling rate, decrease of the grain size and the secondary dendrite arm spacing (SDAS). Owing to the change of heat inputted and laser energy with the different wave of laser as well as the parameters of PW laser such as pulse frequency, the orientation of the grains, which is closely related to the heat source movement, would be affected.
(4) By optimizing the parameters of PW laser, the hardness would improve because of the decrease of grain size, the formation of strengthening structure and hardening phase.
(5) Compared to the CW laser, the PW laser, which can result in the high cooling rate, can increase the crack resistance. The increase of duty cycle is not conductive to preventing from the propagation of cracks because of the increasing of dendritic spacing and then bigger driving strain created by the shrinkage and deformation. In addition, the residual stress increases with the reduction of pulse length and duty cycle.
(6) By using the low-pulse frequency of PW laser, the refined solidified microstructure can be obtained that is beneficial to the corrosion behavior. the micro-porosity, which is also an important factor affecting the corrosion preformation of depositions, would decrease with the decrease of pulse frequency and thus the increase of cooling rate that contributes to the improvement of corrosion behavior.

## 9. Future Perspectives

LMD is, by far, one of the most extensively employed AM technologies. The pulsed LMD technology is expected to maintain and even increase the impact on laboratory investigation and industrial application. The reason behind the continuous impact is without any doubt related to the improved controllability of the LMD process, solidification of molten pool, and then the mechanical properties of deposition by using the PW laser, but also to the recent developments of pulsed LMD using a large variety of different materials, such as Fe-based alloy, Co-based alloy, Ni-based alloy, titanium, and so on. Despite the advancements, the pulsed LAM still have some drawbacks, such as residual stress caused by high cooling rate and high challenge of thermal distribution controlling, that require consideration. This review also addressed the relevant limitations and analyzed the solutions reported in the recent literature.

The optimization of PW laser parameters, the analysis of temperature field by PW laser, and the improvement of mechanical properties including hardness, wear resistance, corrosion, and so on, are currently the center of multiple investigations about pulsed LAM. However, the application of pulsed LAM process for metal materials which have a higher melting temperature, such as high temperature alloy, has a good foreground because the PW laser can produce a higher peak power during the average power is same compared to the CW laser. Furthermore, the PW laser is a promising method to improve the mechanical properties and surface finish of metal parts owing to the better capacity of controlling over the temperature of molten pool. In addition, a smaller molten pool can be created by using the PW laser that is ideal for the fabrication of thin-wall part. Taking into consideration all these facts, it can be concluded that a leap forward for the pulsed LAM, which is mainly investigated in a laboratory at present, could be prompted to the industrial application in the future.

## Figures and Tables

**Table 1:** Summary of materials used in pulsed laser material deposition.

Substrate	Powder (Particle Size)		Duty Cycle	Pulse Frequency Hz	Peak Power W	Scanning Speed mm/s	Powder Feed Rate g/s	Refs.
Low carbon steel Mild steel A36 mild steel 316 L	Fe-based (54~150)	TiC-VC reinforced Fe-based powder	0.65~0.95	5, 50, 500, 4500, 5000	850, 1000	4		[25,26]
		high-nitrogen steel	0.5		1200–1800	5–11	0.167	[27]
		316 L	0.5	8.3, 12.5, 20, 25, 50, 100	450, 1000	6	0.14, 0.078	[28,29]
		Fe-20 wt.% Al	0.15~0.6	30, 60	1090, 1140, 2300	1	0.033	[30]
Ti-6Al-4V Low carbon steel	Ni-based (43~150)	Inconel 718	0.4,0.7,1	10, 20, 100, 1000	300, 600, 857, 1500	4.6	0.358, 0.586, 0.674	[31,32]
		K447A	0.3–0.8	100	216–720	4	0.09	[33]
ST14 plain carbon steel AISI420 Ferritic steel Inconel 713 Low carbon steel Copper alloy AISI 4135	Co-based (45~180)	Stellite 6	0.09–0.48	15, 40, 60	1110, 1330, 1530, 1660, 2220	3~9		[34,35,36]
		Stellite 31	0.16–0.32	16, 20		2, 4, 6		[37]
		WC–12 wt.% Co	0.09~0.63	20~90		1~10	0.05~0.13	[38]
		Co-based alloy	0.03~0.13	40, 60, 92	300, 330, 360, 390	5~11	0.22, 0.42, 0.62	[39,40,41]
Ti Ti-6Al-4V	Ti-based (45~75)	CoCrFeNiNbx/CoNiTi/CoCrNiTi/CrFeNiTi/CrNiTi	0.04–0.1	6, 20	1000	3,8		[42,43,44,45]
		Titanium	0.08–1	40, 60, 100	300–800	6.7, 10		[46,47]

**Table 2:** Cooling rate around 1370 °C and dendrite spacing of K447A alloy deposition [33].

Duty Cycle	Cooling Rate (°C/s)		Secondary Dendrite Spacing (μm)
	PW Laser	CW Laser	
0.3	24,333	3570	4.87
0.4	5840	2393	5.94
0.5	3133	2169	6.32
0.6	2053	1709	6.68
0.8	1557	1557	6.86

## Data Availability

Data is contained within the article.
